# Chronic hepatitis and HIV risks amongst Pakistani migrant men in a French suburb and insights into health promotion interventions: the ANRS Musafir qualitative study

**DOI:** 10.1186/s12889-020-09459-x

**Published:** 2020-09-12

**Authors:** Johann Cailhol, Nichola Khan

**Affiliations:** 1 Infectious diseases department, Avicenne University Hospital, 125, route de Stalingrad, 93007 Bobigny, France; 2 Laboratoire d’Educations et des Pratiques de Santé, Université Paris 13, Bobigny, France; 3 Centre for Research in Spatial, Environmental, and Cultural Politics, School of Applied Social Science, University of Brighton, Brighton, UK

**Keywords:** Migration, Pakistan, Europe, Hepatitis C, HIV, Risk, Vulnerability, Qualitative study, Health promotion

## Abstract

**Background:**

Seine-Saint-Denis is a deprived departement (French administrative unit) in the North-East of Paris, France, hosting the majority of South Asian migrants in France. In recent years, the number of migrants from Pakistan, which has a high prevalence of hepatitis C globally, increased. As a corollary, this study addressed the high proportion of Pakistani patients in the infectious diseases clinic of a local hospital, diagnosed with hepatitis C, but also hepatitis B and Human Immunodeficiency Virus (HIV). It explored genealogies and beliefs about hepatitis and HIV transmission, including community, sexual and blood risk behaviours. The aim was to understand the ways these risk factors reduce or intensify both *en route* and once in France, in order to devise specific forms of community health intervention.

**Methods:**

The study took place at Avicenne University-Hospital in Seine-Saint-Denis, and its environs, between July and September 2018. The design of the study was qualitative, combining semi-structured interviews, a focus group discussion, and ethnographic observations. The sample of Pakistani participants was selected from those followed-up for chronic hepatitis C, B, and/or HIV at Avicenne, and who had arrived after 2010 in Seine-Saint-Denis.

**Results:**

Thirteen semi-structured interviews were conducted, until saturation was reached. All participants were men from rural Punjab province. Most took the Eastern Mediterranean human smuggling route. Findings suggest that vulnerabilities to hepatitis and HIV transmission, originating in Pakistan, are intensified along the migration route and perpetuated in France. Taboo towards sexuality, promiscuity in cohabitation conditions, lack of knowledge about transmission were amongst the factors increasing vulnerabilities. Participants suggested a number of culturally-acceptable health promotion interventions in the community, such as outreach awareness and testing campaigns in workplaces, health promotion and education in mosques, as well as web-based sexual health promotion tools to preserve anonymity.

**Conclusions:**

Our findings highlight the need to look at specific groups at risk, related to their countries of origin. In-depth understandings of such groups, using interdisciplinary approaches such as were employed here, can allow for culturally adapted, tailored interventions. However, French colour-blind policies do not easily permit such kinds of targeted approach and this limitation requires further debate.

## Background

Increasing migrant flows in Europe have prompted estimates concerning the risks associated with ‘imported’ infectious diseases, in order to adapt prevention strategies. For instance, it is estimated that France and the UK have similar rates of contribution by migrants to chronic hepatitis C, as high as 35% [1]. In the UK, studies on hepatitis amongst recently-arrived Pakistanis show a much higher prevalence of hepatitis C than in Bangladeshi or Indian communities, and again much higher among those originating from Punjab, in contrast to other Pakistani provinces [2]. These figures are unsurprising since Pakistan has a high prevalence of Hepatitis C, with a prevalence of 5% in the general population in 2018 [3]. According to the abundant literature on the epidemiology of chronic hepatitis C in Pakistan, the bulk of transmission is constituted by unsafe therapeutic injections [4–8]. By contrast, hepatitis B and HIV are far less prevalent in Pakistan (2.5% in 2010 and 0.1% in 2018 amongst adults aged 15–49, respectively), but HIV is on the rise and at the stage of a concentrated epidemic [9, 10].

France and the United Kingdom (UK) have rather opposed national policies in terms of population data: while the UK systematically categorises ethnicity, France applies a ‘color-blind’ lens to its census [11]. Countries of origin are categorized into sub-continents (e.g. Sub-Saharan Africa, North Africa, South Asia) for organizing data other than immigration statistics. In the field of migrant health, studies on Sub-Saharan Africa (and to a lesser extent from Caribbean Islands) are predominant in France, due to historical colonial and post-colonial ties. Beside, studies on migrants from other areas are in the minority, and those on specific ethnicity and risk of diseases are almost inexistent. Yet the singularity of communities from South Asia in France requires highlighting: their situation differs from that of the UK where these communities are part of the long history of British colonialism and the Commonwealth system and are quite well integrated. In France, Pakistani settlement is more recent (post 1970′), and much less visible [12].

Seine-Saint-Denis, a deprived ‘departement’ (French administrative unit, below region and above district) located in North-East Paris, historically hosts colonial and post-colonial labor migrants from North Africa and Sub-Saharan Africa. Its proportion of foreign inhabitants is the highest amongst French departements (21%) and its South Asian population has increased in recent years. In 2011, the Seine Saint Denis departement hosted one third of immigrants from the Indian sub-continent in France, densely concentrated in 4 districts of this departement [13]. The Pakistani population is the second largest population from South Asia, representing over 6000 official inhabitants [13]. Unofficial numbers might far outweigh this figure, since the International Organization for Migration (IOM) and non-governmental organizations (NGOs) on the Eastern Mediterranean route report a growing number of Pakistanis (mostly men, from Punjab province) [14]. In Greece in 2012, an official figure of 15,000 Pakistani official migrants contrasted with an estimated 60,000 unofficial migrants. In 2014, Pakistan was the fifth country most represented amongst asylum seekers in France, of similar numbers as those from Syria [15].

In concordance with the above European data, Pakistani migrants receiving healthcare in NGOs such as Médecins du Monde were primarily without health coverage (being mostly undocumented) [16]. Correspondingly, there was a spike in chronic hepatitis C referrals at the site of this study, in 2016 and 2017.

In this emergent context of recent Pakistani migrant settlement in France, there is a need for better understandings of hepatitis and HIV transmission, and associated engagement in risky behaviours.

We therefore conducted an in-depth analysis of risky behaviours for hepatitis and HIV amongst a sample of Pakistani migrants. We included HIV, as HIV and hepatitis share common risks factors (as well as some prevention methods) and are tested for together as part of sexually transmitted infections (STIs) screening in France. We sought to identify general knowledge and behaviours pertaining to both infections. We analyzed participants’ behaviours within the wider context of beliefs and knowledge about transmission, their living conditions pre-migration, their journey and living conditions in France, and related mental health status. While mindful that beliefs do not causally determine behaviour, in the case of these participants we started with the premise that they could be linked. Finally, we considered opportunities for community-level prevention in France, together with participants and with community-stakeholders. We strongly propose that in this case, health promotion including prevention interventions, need to be developed in partnership with communities [17].

## Methods

We used the 32-item checklist from the consolidated criteria for reporting qualitative studies (COREQ) as a guide to describe our methods [18]. The check-list is included as additional file 1.

### Study design

The study was qualitative and ethnographic in nature, and named Musafir (Urdu), which translates as “traveler” with connotations of migrant, or one who is separated or exiled from their homeland. It was used to explain the study to participants without conferring a stigmatizing label.

Thirteen semi-structured interviews (one repeated) were conducted between July and September 2018. One focus group was organized in September 2018. In addition, author 2 collected ethnographic data, and attended several meetings with Urdu-speaking general practitioners from the Seine-Saint-Denis neighborhood.

### Recruitment and data collection

Participants were selected from a list of Pakistani patients followed up in the infectious diseases clinic, at Avicenne University Hospital, in Bobigny, located in Seine-St-Denis. The eligibility criteria were: having been born in Pakistan; experiences of living with hepatitis C/B (including those who had been cured) and /or HIV; having arrived in France after 2010, and being willing to participate to the study. The cut-off point of 2010 was chosen in order to take into account the mean time for settling in a country, from 7 to 9 years, during which migrants face hardships and are more vulnerable to risky behaviours [19].

Participants were recruited for individual interviews during follow-up outpatient visits by their clinicians between April and June 2018. Participants were given a choice of interview location. All preferred the out-patient clinic likely due to concerns for privacy and anonymity, a preference for a setting where their hepatitis or HIV status was known and not judged, and where they could avoid potentially embarrassing explanations if friends or acquaintances from the local community saw them. Individual interviews were conducted by one interviewer; a clinician (author 1, MD, PhD), an anthropologist (author 2, PhD), or a clinical psychologist (MSc), assisted by one professional medical translator, between July and September 2018. The clinician and psychologist did not interview participants whose routine care they were responsible for. All interviewers were experienced in leading qualitative interviews. Interviews were conducted in French / English and Urdu, or a mixture of all three, as conversations took their own course. All interviews were audio-recorded. Oral consent was obtained before each interview and recording. At the end of each interview, participants were invited to participate in a focus-group discussion (FGD).

The FGD was conducted in September 2018, after a preliminary analysis of individual interviews. Also present was the same translator who attended the individual interviews. The FGD was conducted in English/Urdu and audio-recorded, after obtaining oral consent from all participants. The overarching aim was to discuss strategies for community-level prevention regarding hepatitis and STIs, as well as health promotion strategies. We opened the FGD by specifying that participants shared one commonality, that being they were Pakistanis with experiences of hepatitis (personal, or via friends, or relatives). There was no disclosure or discussion of personal diagnoses or experiences.

Each participant chose a pseudonym, and full anonymity was observed during individual interviews, which occurred on different days, or at different times of the day, so as to avoid unwanted meetings at the hospital. However, some participants had established friendships through meeting previously at the hospital. They were among those individuals who volunteered for the FGD.

Interviews and FGD were transcribed and translated into English by an independent British company, accustomed to working with medical and social sciences material. Author 2, who speaks Urdu, double-checked the validity of the transcripts against the translations and also the recordings.

The semi-structured interview guide (schedule) was developed for the purpose of this study, using the literature review on risk factors of hepatitis C, B and HIV acquisition (via blood contact- unsafe injections, transfusions, sharing razors or miswak- mother-to-child transmission, intravenous drug use and unprotected sex) as well as factors influencing risky behaviours, before, during and after migration (e.g. increased vulnerabilities from migration conditions, inadequate healthcare, lack of knowledge). The FGD guide (schedule) was subsequently developed, by building on preliminary data analysis. Both schedules are provided as additional files 2 and 3.

All interviewers, as well as the translator were non-Pakistani and non-Muslim. Author 2 is highly knowledgeable in terms of Pakistani society. The fact the interviews were anonymous, conducted in a hospital-setting and by culturally-distant individuals may have served to gain the trust of participants.

### Methods of analysis

**Fig. 1:**
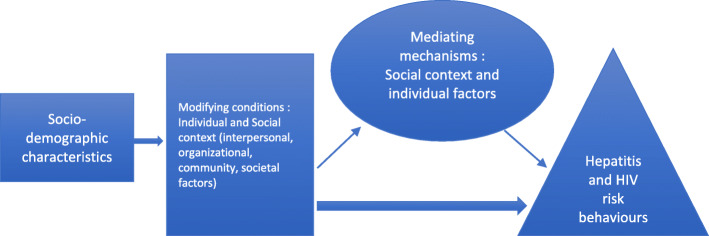
Conceptual framework (own creation), adapted from Sorensen et al. [20], permission for re-use obtained from Elsevier

Notes were taken on attitudes, hesitations, gestures, body languages and tones, and used to interpret data or the lack thereof. Transcripts were analyzed using Nvivo® software, by author 1. The analysis was both deductive and inductive. Framework analysis was first applied in order to deduce exposure to known risk factors to hepatitis and HIV according to the interview schedule, before, during and after migration. Grounded theory was next applied to the overall transcripts, which uncovered emerging and intertwined social and individual factors influencing participants’ behaviours. We subsequently adapted the framework developed by Sorensen et al [20], to describe the influence of the context on individual risky behaviours, in order to propose a terrain for health promotion and prevention work (Fig. 1). Triangulation was performed with the translator and across the three interviewers who represented different disciplinary backgrounds, and also supplemented with field notes and ethnographic observations.

## Results

The characteristics of the participants are presented in Table 1. These socio-demographic data (including the fact that the sample was exclusively constituted of men) largely concord with those of the cohort of Pakistani patients followed-up at Avicenne, and of the IOM study, which described the majority of recent migrants from Pakistan being constituted of single young men from Punjab [14].

**Table 1:** Participants’ characteristics

Participant characteristics		
Participant gender	Male	13
	Female	0
Province of origin in Pakistan	Punjab	13
	Other province	0
Age (years)	Median (extremes)	30 (23–56)
Type of infections	HCV	9
	HIV	2
	HBV	1
	Co-infections HCV-HIV	1
Status	Single	5
	Engaged, fiancée in Pakistan	3
	Married, wife in Pakistan	4
	Married, wife in France	1
Period since arrival in France (years)	Median (extremes)	5 (8–2)
Fluent language	Urdu exclusively	12
	Urdu and French	0
	Urdu and English	1

Most participants entered France undocumented and remained so until they applied for a temporary residence permit for medical reason. In some cases, participants had applied unsuccessfully for asylum, and were undocumented. Local South Asian doctors in the Seine-Saint-Denis departement had referred most participants, symptomatic, to the hospital.

### Life-course trajectories and hepatitis /HIV risk factors

#### Before migration: poverty and myths

The pre-migration context of participants was dominated by hardship during childhood and youth: most grew up in rural and poor farming families in Punjab province, attended primary school (some religious, other public); a few were from families owing farms or shops, but were precipitated into economic decline. Some participants had family members living with hepatitis C. Although many were aware that HCV and HIV were dangerous, they had little clue about their shared transmission routes. They were not knowledgeable at all about HBV. They unanimously thought that dirty water was the main HCV transmission route, together with dust, and that hot chillies could reactivate the virus. The few participants who had been tested for hepatitis C before migration did so exclusively because of “yerkaan” (Punjabi), meaning jaundice, a visible symptom, or “kala yerkaan” meaning a hot liver. Participants all believed “yerkaan” could not affect a child or young adult and that they could not carry the infection without any symptom.

They considered HIV more dangerous than HCV, and associated its transmission with female sex workers. They discovered, once in France, that unsafe injections could be a transmission route, especially for the 3 viruses. Many participants had received therapeutic injections, especially for fever and fatigue. Pakistanis indeed have a strong belief in the efficacy of therapeutic injections [21] and quack doctors are widespread [8]. Participants all highlighted the divide between the expensive health care provision in Pakistani private hospitals, where good doctors practice (‘only rich people get treated at hospitals’, participant 1), and the cheap but unreliable care in villages offered by so-called quack doctors (‘we don’t have any other option, we don’t have any other doctor’, participant 2).

Participants had occasionally patronized street barbers who worked with razors without disposable blades. A number of participants additionally reported having witnessed or being involved in sexual activities between boys (starting around age 13 at single sex school), relations which continued subsequently into adulthood. Some had heard of child abuse (i.e. men abusing boys) and qualified it as being quite frequent. Mutual sex between male adolescents was discussed as a commonplace practice in rural communities and, if not typically spoken about openly, did not necessarily represent a shameful or unwanted practice. By contrast, “Men having Sex with Men (MSM)” was viewed as separate from heterosexual marriage which all aspired to, and a gay identity strongly was refuted.

“An adolescent and a man, it is not a gay relationship, an adolescent is not a man” (FGD).

Injecting drug was also reported as being frequent in Pakistan, but no patient disclosed having engaged in it.

#### The journey: long and traumatic

The lure of migration initially tempted participants as an exciting opportunity to mitigate poverty and support their family, escape dangers, or simply undertake youthful adventure with friends. Most young men knew of enviable others in these small communities who had migrated to Europe and benefited their household via remittances. Some leaving Pakistan did so upon official invitation from a relative or friend already settled in Europe (mainly Italy), allowing legal entry. They then became undocumented upon expiration of their 3 months Schengen visa. The principal migration mode was via an ‘agent’ (trafficker), at substantial cost to the family. All our participants who took the land route via the Eastern Mediterranean route, were affected by violence: psychological fear of being arrested, or fired on by army personnel; being held to ransom by smugglers; seeing their travel companions shot, or abandoned; physical exhaustion after walking for days, hunger, thirst, being cramped; as well as sexual violence. The journey to Europe typically took months and sometimes years, with several being deported and re-smuggled across borders multiple times before reaching France. Many stayed in transit countries such as Iran, Turkey, or in other European countries such as Greece or Italy for years, living in multiple places or migrant camps, and taking small jobs where possible. One participant engaged in transactional sex with men in exchange for accommodation and work, this being fairly common, whereas other participants reported having witnessed fellow countrymen *en route* being forced into sex. All participants shared razors and crockery during the land-journey, due to an extreme lack of privacy.

#### Life in France: precarious and clandestine

A sense of temporal and spatial distance prevailed among Pakistani communities in Seine-Saint-Denis: none of the recently arrived participants mixed with older generations of Pakistanis in France, who had settled in other areas. Almost without exception, participants shared small rooms with other recently arrived men, also from Punjab province in Pakistan. Such living arrangements are common among South Asian migrants in Europe. Except for two, all participants had unstable precarious lives and worked in short-term contracted jobs. They mostly worked as painters on building projects, or electricians. Low-paid labour and complex dependencies on their ‘bosses’ mean that many Pakistani migrants in Europe work many hours daily, for 6 or 7 days a week, for months on end, without respite or holidays [22]. Those who worked together and cohabited inevitably formed stronger ties—taking turns to shop and cook Pakistani food, share bills and chores. They admitted at times sharing toothbrushes and razors to shave their face, armpits and genital areas, customary shaving practices for Muslims. Once diagnosed with hepatitis and/or HIV and advised, participants became cautious about not sharing their personal belongings. Most disclosed their hepatitis status, and their roommates were sympathetic to them, since in Pakistan hepatitis and symptoms such as jaundice are understood to be common national conditions.

Due to suspicion over being arrested, language barriers, and the imperative will to work, they barely had any leisure time. Most were single. A few disclosed having girlfriends (non-Pakistanis). Others, longer-settled, went to sex-workers (mostly engaging in unprotected intercourse). A few did not engage in any sexual activity. Some participants reported having ‘heard about’ men engaging in same-sex in France (Pakistanis with Pakistanis, or with Arabs), and one specifically knew friends who had done so. Some were pragmatic:

“If there are no women, a man may have sex with another man, just to fulfill his needs” (participant 1).

Drug users appeared uncommon in the community, even though few participants mentioned having heard of Pakistanis using/selling heroin in Seine-St-Denis. None declared having used drugs themselves. By contrast, barbers seemed to be a significant potential area for transmission risk, especially in the wider community (where many Sri Lankan and Bangladeshi barbers run small shops). Areas such as Strasbourg-St-Denis in Paris (hosting a large North Indian community) and La Courneuve were, during the FGD, cited as places where barbers may not be ‘safe’. These data were supported by some ethnographic observations of barbers who used the same blade for consecutive customers. One participant mentioned having received dental care from an informal Indian dentist in a workers’ hostel. These findings bear unexpectedly on implications for the transnational mobility of informal and unregulated healthcare practices between South Asia and France.

### Individual and social factors influencing behaviours

We interrogated here individual and social factors influencing behaviours in light of the Sorensen model (Fig. 1).

#### Transnational societal norms and taboos

Even while living in France, there was an overall sense of fear of authorities, and sense of shame or outright denial when discussing behaviours that contravened Pakistani societal norms. For example, the majority of our participants admitted MSM practices being fairly common between Pakistanis, in Pakistan or abroad, but that these practices were ‘invisibilised’ through not being discussed, leading to contradictory statements in our interviews. One participant insisted ‘It doesn’t happen in Pakistan, it’s against Islam’. Even ‘talking about MSM’ was considered taboo outside of very private, personal, male friendships:

“In Pakistan, you won’t say something like that – man [having sex] with man- you can say openly here, but not there in Pakistan” (participant 3).

The few who spoke without stigma about MSM practices were younger, whereas older participants were judgmental and adamantly affirmed the illegal, morally prohibited (haram) nature of MSM practices. Moreover, hierarchy was very much respected, even in France, and some intimate topics were unspoken between generations.

“Elders won’t talk about their ‘girlfriend’ to younger ones” (FGD).

Discussions around women were permitted (between same aged men), normalised in a male-dominant model of society, whereas disclosing MSM experiences could risk their families discovering it.

“About [sex with] women they [Pakistani friends] make jokes but about men they are very cautious, they don’t say because they know that this will go to Pakistan and it will be a big mess.” (Participant 4).

Participants were cautious to not shame their family by revealing non-conformist sexual practices, notwithstanding the geographical distance: most of them awaited their family in Pakistan to choose a wife, but reported transnational sexual and romantic relationships, including adultery, as being common. The public admission of non-marital sex this implies is deeply shameful in the family context.

Overall, there were strong pressures for prosocial behaviours such as respect for generational hierarchies, familial and cultural norms. These meant socially prohibited and transgressive behaviours in participant’s private lives were hidden. Yet Islam remained prevalent mostly as a moral discourse, rather than a strict set of rules to follow, while seeming to shape behaviours and attitudes.

#### Community and family ties

Our participants were very much embedded within Pakistani migrant communities in France. They mixed largely with other Pakistanis from Punjab who were recently arrived, due to, *inter alia*: their common language (Punjabi), pre-existing networks which provided access to employment and shared accommodation, and their ethnic/regional commonalities (reflecting the transnational adaptation of regional hostilities and ethnic loyalties in Pakistan, in the French context). While these communal bonds of friendships and sociality operate in their favor, they also potentially lead to hepatitis and HIV transmission: via the sharing of personal belongings (razors, syringes); via community-born risks (barbers, informal medical care); or via MSM practices between friends. These communities also offered forms of identification and support regarding their obligations to their community in Pakistan, which allowed them to leave their country, but under certain conditions such as sending remittances. Family ties are of enormous importance and this debt ties them, as with many first-generation migrants or refugees to Europe to their families transnationally. Yet in Pakistan, Ahmad argues that migration constitutes a natural inclination amongst young men and frames the desire to migrate as a condition of masculinity amongst young Pakistanis [22].In terms of sexuality, he introduces the notion of “melancholia” in desexualized “dead bodies” working “invisibly” in order to send remittances informing Western representations of undocumented migrants [23]. Invisible sexualities apply too to our migrant sample whose sexual desires are effaced or hidden because their families might discover they have sex lives and chastise them; because of culturally-shaped personal beliefs about taboos, and morally reprehensible behaviours [24]; because of a habitus of secrecy in a country with post-colonial history of Islamic state nationalism becoming increasingly right wing and conservative [25]; and because of the extreme restrictions and hard labour conditions migrants and refugees face in Europe [26], combined with the hidden shame of their diagnosis (especially if HIV).

Nonetheless, agency should not be under-estimated and we note the importance of not reproducing Orientalist tropes about the oppression of Islam. The interviewees all hoped that the study would help their local community in France. This has implications in terms of health promotion: certainly behavioural interventions need to tackle social and culturally-specific mediators [27].

#### Poor mental health status

Participants’ overriding mental health was characterized by pervading anxiety (referring to poor sleeping quality, rumination, no hope in the future, and fears), deriving from multiple sources, some medical: HIV status which could not be disclosed; hepatitis C hypothetic survival in the body, and its potential for reactivation (based on false beliefs such as via eating hot chillies); infertility due to HIV or hepatitis, and transmission risks to a future spouse; the risk of becoming re-infected when back in Pakistan. Many suffered from traumatic reviviscences of their migration journey. Participants in our sample reported normalized experiences of violence (including sexual) during their journeys to Europe, further constituting a risk and poor mental health (in addition to risks for hepatitis and HIV). Our data indicate far worse conditions on the Eastern Mediterranean route than those from the IOM study [14], which reported ‘only’ 20% incidents of violence perpetrated on a sample of Pakistanis. This could be due to either our sample being constituted by particularly vulnerable migrants, or to an underestimation of the IOM study.

None of our participants reported they drank alcohol, when asked by their physician. However, several reported having used alcohol before being diagnosed. Above all, their priority was to work and earn money and send remittances to family, and being sick or unable to work due to illness was a significant source of anxiety.

Ongoing legal status and paper issues were central for most participants, and produced much mental tension. Most navigated between rounds of asylum and temporary residence permit for medical reason applications, and had undocumented status.

Almost all our participants suffered from precarious unstable living conditions after migration to France, which constitutes per se a determinant of health and disease acquisition, such as hepatitis and HIV [19].

Moreover, poor mental health, which was prevalent in our sample of Pakistani, is a mediating factor affecting, for instance, self-efficacy in adopting non-risky behaviours. Certainly mental health needs should be accounted for in the design of health promotion and prevention interventions.

## Discussion

Our study suggests that our sample of Pakistani participants, living with hepatitis and/or HIV, might well have acquired their viruses in Pakistan (via unsafe therapeutic injections, sexual abuse, drug use), but also *en route* (via sexual violence, sharing personal belongings). Once in France, risks of hepatitis (and HIV) transmission seem to persist, in the form of hidden MSM practices, informal dental/medical care and unsafe barber’s shops. Such risks could constitute a non-negligible source of transmission, though extremely difficult to uncover, according to our findings and the literature [28–32]. Indeed, homosexuality has been criminalized in Pakistani law since colonial times, and ‘pathologically high levels of discrimination and contempt towards sex workers, injecting drug users’ exist [33]. In parallel, some reports from Pakistan, as well as academic studies, indicate that sexual relationships between men might be normalized and occur at quite high frequency, although be hidden by a veneer of hypocrisy [28, 31, 32, 34, 35]. Pakistan has a high prevalence of HIV/hepatitis amongst hidden MSM [36]. Fortunately, there are increasing advocacy initiatives in Pakistan for HIV prevention, and MSM specific-interventions [37, 38].

Finally, one important finding was that barbers in the Avicenne hospital neighborhood were potentially unsafe, as are many barbershops in Pakistan [39]. This provides evidence of the transnational mobility of informal, unsafe hygiene practices between low- or middle-income settings and marginalized areas of high-income setting.

### Implications for health promotion interventions

Our findings hence have implications in terms of hepatitis and HIV prevention and health promotion. Health interventions will not be achieved in this migrant community without a strong involvement of the community itself [17] and a change in several practices and norms. Actions proposed hereby derive from discussions with interviewees and from the FGD, and are embedded in the Sorenson model [20].

First, there was a convergence towards the need to establish an early diagnosis for all who might silently carry these viruses within the community. Testing should be an outreach action. Following a mapping exercise, we propose several places Pakistanis typically work in wide Paris suburbs. This outreach work would be open to all communities, especially South Asian Muslim communities in the targeted areas, in order to not stigmatize Pakistanis. This outreach campaign could also be coupled with a knowledge-attitude-practice survey, adapted to cultural norms (e.g. stigma, discrimination and a reluctance to talk openly about sex, and MSM) and literacy rates. In order to enhance the acceptability of the testing campaigns, we propose also a series of awareness campaigns at the Pakistani mosque (located in the suburb close to the hospital) during celebrations, with the support of the mosque association and Urdu-speaking local doctors. A growing body of literature, especially in the UK and USA, mentions the usefulness of including faith-based organizations in the health promotion interventions, given the strong collectivism and faith-based values of South Asian communities [40–42]. We did engage with the local Pakistani mosque leaders, with the help of participants, and agreed to work in partnership on future collaborations. However, we also recognize that faith leaders are also those most likely to hold conservative, stigmatizing values and the limitations of this approach, which can only be partially successful.

Secondly, community education on prevention and sexual health promotion is essential but needs to be culturally-specific, given the importance of transnational social norms [43], regarding sensitive topics such as homosociality, drug use and male sexuality. Based on the FGD in which full anonymity came out as a prerequisite for deploying such education, we propose using social media or a website in Urdu, administrated by an Urdu-fluent health professional. The media would include information on sexual health promotion and prevention, as well as a forum for online questions & answers and discussion. Participants would benefit from an entire anonymity, allowing discussion around sensitive topics. Social media is increasingly used in sexual health promotion, given the sensitivity of the topic, and evaluations, though still rare, are positive [44].

Thirdly, in parallel to this community-level action, individual empowerment and stigma reduction should be sought, via increasing literacy rates, human rights awareness, and support around access to healthcare, especially for STIs and hepatitis, and mental health. We propose for this purpose, to train people from Pakistan or from Pakistani descent, fluent in both Urdu and French, to become community health worker. Community-health workers are still not a well-defined category of workers and are defined by their competence rather than their training [45]. They owe cultural competence, are trusted by the community, and are able to empower the community by raising their health literacy and *in fine* empower them [46]. Ipso-Care is an example of well-functioning NGO of psycho-social counselors from the community who work for the community, which could be replicated here [47].

Fourthly, we propose to conduct a survey on knowledge-attitude-practices in occupational health, in the barbershops held by South-Asian communities in the hospital neighborhood and other similar neighborhoods. In Ghana for instance, a similar survey revealed enormous gaps in knowledge and related risky behaviours towards blood-borne viruses [48].

These four types of actions were proposed to the local health authority of Seine St Denis departement, and funding was secured for their implementation in the coming year. These interventions will be implemented in a community-participative way, and will be evaluated.

### Limitations

Our study has some limitations. Our sample is not representative of all Pakistani migrants, but rather represents Pakistanis affected by chronic hepatitis and/or HIV, living in precarious conditions in Seine-St-Denis. Our sample does however represent the most vulnerable ones, the undocumented migrants, who are tremendously difficult to study [49], due to fears of the French administration and language barriers. The very mobility of Pakistani migrants on the move mean that contact tracing can only be of limited effectiveness. Moreover, these migrants represent a significant number, given IOM unofficial research on the Eastern Mediterranean route. Our sample size is small, but continuous analysis during data collection showed that data saturation was reached. Patients seen subsequently at the outpatient clinic after the study had confirmed many of the findings. It is likely that participants did not disclose everything relevant to transmission risks, especially about MSM practices among their community, in Pakistan, *en route* or after arrival. Given the extreme stigmatization of these events or social prohibition of these behaviours, we interpreted our data with caution. Nonetheless, some specific personal disclosures, combined with emerging literature, lead us to conclude that some neglected risks might be well present in this community.

## Conclusions

In our sample of recently-arrived Pakistani migrants, essentially constituted of young single men living with hepatitis and/or HIV, persistent risky behaviours towards hepatitis and HIV in France relate primarily to marginalization, promiscuity and poverty, enhanced in turn by poor mental health, lack of education, low literacy, unequal and differentiated access to health care and denial of risks due to social and cultural norms. Future research will include a phylogenetic analysis of a larger group of patients with chronic hepatitis C. This would further assist in more precisely assessing temporal-spatial patterns of transmission and locating whether transmission occurred in Pakistan, *en route* or in France. Further ethnographic research onto informal medical and dental care practices in migrant communities might mitigate further rising risks of unforeseen transmission.

Our study identified many mediators, which could be used to promote health and to change behaviours. Those mediators are a collectivist approach to community, with a strong sense of community. There is also space for change, as stated by one participant: “we will never be able to talk about condoms in Pakistan, here in France we can” (FGD). This points to individual agency as a positive force.

However, it is important to note there are also substantial limitations imposed by the isolation and non-integration of a migrant community that perceives itself as a community of temporary workers who will most likely return to Pakistan, or move on within Europe. The extent to which it is possible to develop forms of health promotion that are likely unfamiliar to migrants, albeit culturally sensitive and specific, without for example improving the very poor living conditions of this community is a realistic concern. These should address cultural perceptions of both individual and social risk [50] for example, migrants’ fears of not being able to work—to have greater take-up.

More broadly, this study points out to the need to go beyond the universalism advocated by the French Constitution, and dig into some ethnicity-related specificities [51, 52]. The French Republican model, which prevents from applying colour-conscious measures, has been criticized to hide inequalities associated to ethnicity or race [11, 53, 54]. These two words are synonymous of historically painful memories related to world war for French people [51]. However, such memories need to be overcome, in order to not miss opportunities to tackle health inequalities, while being cautious in avoiding stigmatization.

## Supplementary information

**Additional file 1.** Consolidated criteria for reporting qualitative studies. COREQ guidelines checklist applied to our paper.

**Additional file 2.** Interview guide Musafir study. Semi-structured interview guide developed for the purpose of this study.

**Additional file 3.** Focus group discussion guide. Focus Group Discussion guide developed for the purpose of this study.

## Data Availability

Authors are not able to share their raw data since participants disclosed very sensitive information. Participants were told that the transcripts will be read by NK and JC only, and this prompted their trust. However, authors are willing to share codes generated from the data on request (to the corresponding author).

## Abbreviations

ANRS: Agence Nationale de Recherches sur le VIH/SIDA et les hépatites virales (French Agency for Research on AIDS and Viral Hepatitis)
COREQ: Consolidated criteria for reporting qualitative studies
FGD: Focus-group discussion
HBV: Hepatitis B virus
HCV: Hepatitis C virus
HIV: Human Immunodeficiency Virus
INSERM: Institut National de la Santé et de la Recherche Médicale (National Institute for Health and Medical Research)
IOM: International Organization for Migration
MSM: Men having Sex with Men
NGO: Non-Governmental Organizations
STI: Sexually Transmitted Infections
UK: United Kingdom
USA: United States of America
