# Supplementary material for: Chronic hepatitis and HIV risks amongst Pakistani migrant men in a French suburb and insights into health promotion interventions: the ANRS Musafir qualitative study
Source: BMC Public Health. 2020 Sep 12;20:1393. doi: 10.1186/s12889-020-09459-x (PMC7488669; doi:10.1186/s12889-020-09459-x)
Supplement: Supplementary file 2 — Additional file 2. Interview guide Musafir study. Semi-structured interview guide developed for the purpose of this study. [file 12889_2020_9459_MOESM2_ESM.docx]

Interview guide Musafir study

Introduction:

aim of study

who am I (the interviewer)

agreement to be recorded while stressing the fact that all what is said is confidential and anonymous

**Part 1: mini-biography around migration**

Please tell me about your life (childhood, family, major events, past and present, Pakistan, Europe). Start wherever you like, but do include as much detail and information as you can. It is not important to stick to chronological time although you may like to. I won’t interrupt you as you speak or stop you but I will be listening carefully, as much as I can. Afterwards I will ask you questions about your story, but also questions about things you might not have mentioned. Apologies for any repetition due to language issues. You do not need to say things twice if you have already spoken about them, unless you wish to elaborate. *Following questions are only to be asked if no spontaneous reference to it.*

- Before migration

Where are you born, where did you live, school attendance, familial context

- Migration

When did you decide to migrate and why?

What was your initial final destination?

When did you really leave Pakistan, how and with whom?

What were your plans for the future when you left? (e.g. wedding, family life, did you plan to return)

Which countries did you get through and how did you live / how did you pay for travel expenses? Did you work in any of these countries? If yes, to be probed.

How did you communicate with your family?

- After arrival in France

When did you arrive in France? Whom did you live with since the arrival, and where?

What were and currently are your permits statutes and health care access?

Do you have an income? How do you communicate with your family?

**Part 2 Representations (personal and community), knowledge around the chronic condition (HIV or hepatitis B/C), and relations to mental health, religiosity, sexuality, as well as their evolution over time**

Let’s talk now about the condition for which you have a follow-up here in Avicenne (quite medical first and then to be enlarged)

When were your first diagnosed, in which context and what was your reaction? Did you tell anyone in your surroundings? Why?

What did you know about this condition at the time of diagnosis? Do you remember when you heard about this condition for the first time?

Transmissions rates are high in Pakistan, especially for hep C without precisely knowing the origin. Modes of transmission are the following: *Different modes depending on the condition to be cited here*

Would you recall which type of exposure you might have had, in Pakistan or during migration or after arrival? Is there any of these risk factors (expositions) which currently continue in France? To probe

Do you think your condition could be related to your journey or your living conditions here?

to be introduced with precaution if no spontaneous reference to it: MSM seem to be usual before marriage, was is the case for you? Did you experience any violent sexual assault or un-agreed sexual intercourse in your life?

Tell me about your everyday life?

How do you feel about these diagnoses you have? What is your relationship to them? The medical diagnosis is one explanation- but do you have other ideas on how you came to be in this condition, or how to explain it? What words (Urdu) can describe how you feel? Can you explain them in more detail?

What has changed in your key relationships since you received your diagnosis? Do you have family support now? What do people say in Pakistan about HIV/Hepatitis - are things different in France?

Do you believe in jinns? Jinn possession? In God? In fate? In dreams? Give examples. Does your religion give you a psychological support? If yes how? How do you think the diagnosis of your health condition and/ or your migration impacted on your “religiosity”?

How do you live your sexuality / masculinity and how your condition and psychological well-being did affect them? Could you relate those changes to migration, and to your current community?

How do you feel about your life? What is its purpose? What do you feel about death? How do you endure in this incredibly difficult situation? What are your hopes for the future?

**Closing remarks**

Do you accept to be recontacted?

Do you accept to take part to a focus group around findings feedback and building a community-level education program?

Thank you very much for your time
