# Supplementary material for: Chronic hepatitis and HIV risks amongst Pakistani migrant men in a French suburb and insights into health promotion interventions: the ANRS Musafir qualitative study
Source: BMC Public Health. 2020 Sep 12;20:1393. doi: 10.1186/s12889-020-09459-x (PMC7488669; doi:10.1186/s12889-020-09459-x)
Supplement: Supplementary file 3 — Additional file 3. Focus group discussion guide. Focus Group Discussion guide developed for the purpose of this study. [file 12889_2020_9459_MOESM3_ESM.docx]

**Focus group discussion guide**

Thank you very much for coming and taking part to this discussion group.

There are some commonalities between all of you, which are your country of origin, your region (Punjab), a migration to Europe and eventually in France in Seine St Denis. You also all have some linkage with hepatitis C, some are or were affected, whereas others have their family member or relatives affected.

As you all understood, the aim of our study is to get deep insight into the recently arrived Pakistani community in Seine St Denis. Because Pakistan has the second highest prevalence of hepatitis C, we believe that there are opportunities, at community level, to build prevention and screening program for hep C. We also would like to take the advantage of this program to add messages on STIs, as many of the recently arrived migrants are men, single.

You all took part to face to face interview and we would like now to go further down the line and create a debate amongst you.

We will ask one question at a time, and we will moderate the discussion that will occur between yourselves.

We would the discussion to be recorded, so as to not lose details or get wrong messages. The record will only be shared between researchers, and will be deleted afterwards. For this purpose, we will ask you to not disclose your names here.

**Part 1 about Pakistan and migration**

How would you compare the idea you had about Europe before leaving Pakistan and what you are experiencing now?

In general, would you say that life is better in France for you now?

pre-existing contacts and connections here, and how these work transnationally

How do you feel about your migration journey?

How did it change you and your values?

What are the relationships here with other non Pak communities and with Pak community (older generation or recent migrants)? Why are you all from Punjab?

what kinds of friendships do people develop on the journey to Europe, and once here. How do these help people becoming settled, finding a place to stay. What sorts of relationships are made between Pakistanis cohabiting? Do Pakistanis here only live with fellow Punjabis or with Muslims from other countries, or Pakistanis from other backgrounds?

Do your friends mostly live alone or with others? Tell something about those living arrangements.

types of male sociality and friendships developed en route and in/across Europe once they're here

Are the friendships made here stronger/more permanent or temporary compared with Pakistan?

How is Europe imagined from Pakistan- and how does the picture you formed before coming compare with your experience in Europe?

Are Pakistanis still coming to Europe and if not why not?

**Part 2 about hepatitis C (non sexual routes of transmission)**

Yerkaan- (jaundice); kala yerkaan (black jaundice) Jigar ki garmi (heated liver)- Hot and cold- in the interviews some of you understood Hep C causes and symptoms in terms of hot and cold. Please elaborate on what hot and cold mean in regard to illness. What other kinds of illnesses are hot and cold

All Pakistani patients with Hep C in this hospital are from Punjab.

Can you give an explanation for this?

- in the Pakistani community in France Hepatitis C might be well acquired in Pakistan, but the transmissions dynamics might continue if risk factors persist.

For the following risk factor (community and healthcare related), could you discuss it could happen here in Pakistan, during migration or in France, we will talk later about sexual transmission

- Blood transfusion with non sterile material
- Injectable drugs with non stérile syringes
- Intravenous drug injection
- Sharing razors
- barber shops
- Miswaq sharing

**Part 3: sexual transmission: STIs including hep C**

What are the infections that you know are transmitted by sexual intercourse?

What sexual imaginaries of Europe do people hold in Pakistan- how do they differ from the reality? Is the reality disappointing?

How do you find space for your personal intimate life?

What do people think about those who live with girlfriends- it's ok or shameful (to be hidden)?

Some of you told us about common MSM practices in rural Punjab. This is also something we found in the research literature.

- adult-to-child (Coerced or willing)
- Equal, MSM-not gay, “married” or girlfriend
- MSM-gay

Prompts: family, schools, workplaces, for favors, exchanges

Do you know of young men here engaging in transactional sex (for money, favours, gifts, accommodation etc.).

Is there is a difference between the casual sexual relationships formed between young men, and being gay? Are they the same- if not, how are they different? Would you feel happy to know others know you have or do engage in MSM?

Religiosity

We know that the religion does not allow any sexual relationship without being married. Most of you are single and we would like you to discuss how you manage this.

Part 4: prevention

In community in France, where do you think the prevention could take place

- Mosque
- Chantiers
- Restaurants,
- Snacks,
- Phone and internet shop
- Market,
- Grocery
- Barbers

What are your feeelings about condoms?
